# Predictors of the short-term responder rate of Electroconvulsive therapy in depressive disorders - a population based study

**DOI:** 10.1186/1471-244X-12-115

**Published:** 2012-08-17

**Authors:** Axel Nordenskjöld, Lars von Knorring, Ingemar Engström

**Affiliations:** 1 Department of Psychiatry, University Hospital Örebro, Örebro County Council, Örebro, Sweden; 2 Department of Neuroscience, Psychiatry, Uppsala University, Uppsala, Sweden; 3 Psychiatric Research Centre, School of Health and Medical Sciences, Örebro University, Örebro, Sweden

**Keywords:** Electroconvulsive therapy, Major depressive disorder, Treatment outcome

## Abstract

**Background:**

The aim of the present study is to investigate the responder rate of Electroconvulsive therapy, ECT, in clinical routine work and to define clinical characteristics predictive of response to ECT. The main hypothesis is that the responder rate of ECT might be lower in clinical routine than in controlled trials.

**Methods:**

This is a population-based study of all patients (N = 990) treated with ECT for depressive disorders, between 2008–2010 in eight hospitals in Sweden. Patients with Clinical Global Impression-Improvement scores of 1 or 2 (much improved) within one week after ECT were considered responders to ECT. The predictive values of single clinical variables were tested by means of chi-squared tests and the relative importance was tested in a logistic regression analysis.

**Results:**

The responder rate was 80.1%. A higher proportion of older patients (>50 years) responded (84.3% vs. 74.2%, p < 0.001). Psychotically depressed patients responded better (88.9% vs. 81.5% for severely depressed and 72.8% for mildly depressed, p < 0.001). There were no significant differences in responder rates between patients suffering from bipolar, first or recurrent major depressive syndromes, or a depressive episode of schizoaffective disorder. Patients with personality disorder had a lower responder rate (66.2% vs. 81.4%, p < 0.001). Also, outpatients had a lower responder rate (66.3%) compared to inpatients (83.4%, p < 0.001). In the logistic regression analysis, inpatient status, psychotic symptoms, absence of schizoaffective disorder and older age were independent factors associated with response to ECT.

**Conclusions:**

This study focuses exclusively on the short term responder rate with ECT in clinical practice. Similarly to results from controlled trials a high responder rate is reported. Older patients, more severely ill patients, psychotically ill patients and patients without personality disorders had the highest responder rates. Inpatients may have better outcome with ECT than outpatients.

## Background

Electroconvulsive therapy, ECT, is an effective treatment for severe forms of depression, such as psychotic or catatonic depression. ECT has also been recommended in less severe forms of depression after pharmacotherapeutic failure [1]. The efficacy of ECT in severe depression is demonstrated to be high in clinical trials with remission rates of 60–70% or more repeatedly reported [2-4].

There may be a gap between the convincing results of ECT achieved in clinical trials and the effectiveness in clinical practice. In an earlier study by Prudic et al. conducted in routinely treated patients, the response rate was 64% and the remission rate was only 30-47%, depending on criteria [5]. One explanation for the gap may be patient selection. Patients with co morbidities are often excluded from clinical trials. If such patients are less likely to benefit from ECT, it may contribute to the divergence.

The ultimate aim of treatment for depression is to achieve remission of symptoms and restored social functioning. However, symptoms from depression most often abate gradually, and a response to treatment is an important first goal.

Presence of psychotic symptoms[3,6], lower degree of prior treatment resistance [7-9] and shorter symptom duration [8,10] are relatively well established predictors of response to ECT [11]. In addition, the CORE group reported depression in old age to be associated with a favorable outcome [12]. In a study from Finland, younger patients suffering from moderate depression and with co- morbidity had a lower responder rate as compared to severely depressed older patients without co-morbidity [13]. However, there are variations in the results and the importances of several factors are debated. In particular the importance of psychotic depression [7,14] and greater initial severity [15] have been questioned. Thus, more data is needed on the importance of predictors of response to ECT.

The aim of the present study is to investigate the responder rate of Electroconvulsive therapy, ECT, in clinical routine work and to define clinical characteristics predictive of response to ECT. The main hypothesis is that the responder rate of ECT might be lower in clinical routine than in controlled trials.

## Methods

### Subjects

In this study, 990 patients treated with ECT for major depression or schizoaffective disorder, depressed type between January 1, 2008 and December 31, 2010 in eight hospitals in the middle of Sweden were identified. Information about the clinical outcome was available for 936 patients. Our data therefore illustrate a population-based cohort treated in ordinary clinical routine.

Inclusion criteria in the study were:

1) Diagnosis of Depressive Episode (F32), Major Depressive Disorder (F33) Bipolar Disorder, depressive episode (F31.3-F31.5) or Schizoaffective disorder, depressive type (F25.1).

2) Treatment with ECT in one of the eight hospitals in the middle of Sweden between January 1, 2008 and December 31, 2010. Each patient was included only with the first treatment series in the period.

Exclusion criteria from statistical analysis was:

1) No Clinical Global Impression – Improvement (CGI-I) data available after ECT.

### Included and excluded subjects

Out of 990 patients treated with ECT during the period, 54 patients were excluded from the response analysis because no CGI-I data were available after ECT. Patients with and without CGI-I ratings after ECT were similar as concerns age 54 years (SD 18) vs 53 (SD 16), sex (57% vs 61% women), diagnoses (76% vs 79% unipolar depression, 19% vs 17% bipolar depression and 5% vs 4% schizoaffective disorder depressed type). The severity of depression among patients with and without CGI-I ratings were mild/moderate 32% vs 37%, severe without psychosis 47% vs 44% and severe with psychosis 25% vs 16%. The proportion of diagnosed co-morbid personality disorders were 8% among CGI-I rated patients vs 11% for patients without CGI-I rating. 19% of the patients with CGI-I ratings were outpatients vs 28% for patients without the ratings. The number of ECT sessions given for patients with CGI-I ratings was 8.0 (SD 3.2) vs. 7.2 (SD 3.7) for patients not rated.

### The Quality register

The data in this study were derived from the Quality register for ECT. Nurses at each site collected information from the patients’ charts. In Sweden, all ECTs are provided in psychiatric hospitals responsible for the treatment of all patients in a defined geographical area. Eight hospitals in the middle of Sweden collaborated in 2008–2010 to report clinical data to the register. The eight hospitals cover a population of 1.5 million inhabitants. Six hospitals started reporting data in 2008, one hospital started in 2009 and one hospital in 2010.

### Ethics

The Regional Ethical Vetting Board in Uppsala approved the study. The patients were informed about the register and accepted registration.

### ECT-treatment

ECT was administered using a bidirectional constant current, brief pulse device. The Mecta Spectrum 5000Q device (Mecta Corp, Lake Oswego, Ore) was used at six hospitals and a Thymatron system IV and a Thymatron DGX device (Somatics, Inc, Lake Bluff, Ill) was used at one hospital each.

Most treatments were unilateral, but in 13% at least one of the treatments in the series was bitemporal and in 4.8% at least one treatment was bifrontal. The mean dosage at the last treatment if unilateral was 0.49 ms (SD 0.14), 73 Hz (SD 23), 7.4 s (SD 0.83), 840 (SD 53) mA and 451 (SD 186) mC. The mean number of ECT sessions was 8.0 (SD 3.2).

Propofol (mean dosage 107 mg (SD 41) or thiopental (mean dosage 306 mg (SD 98)) was used as anaesthetic. Succinylcholine (1 mg/kg) was used as muscle relaxant and glycopyrrolate (0.2 mg) was used as anticholinergic. If no adverse events occurred, ECT was continued until the patients were asymptomatic or the physician judged that the patient had benefitted as much as possible.

### Other treatments

The pharmacotherapy was assessed at the conclusion of the ECT series. An antidepressant drug was used by 87% of the patients. Selective Serotonin Reuptake Inhibitors were used by 36% of the patients, Serotonin–norepinephrine reuptake inhibitors by 22%, mirtazapine by 25%, tricyclics by 7%, other antidepressants by 19%, lithium by 16%, lamotrigine by 10%, valproate by 3%, benzodiazepines by 24%, other antiepileptic drugs by 9% and antipsychotics by 39%. Specific psychotherapy was seldom used but supportive care was provided. The supportive care was more intensive for inpatients.

### Variables and measures

The Clinical Global Impression – Improvement scale (CGI-I) [16] is a scale ranging from 1 = very much improved, 2 = much improved, 3 = minimally improved and 4 = not improved. Experienced nurses who met the patients during the ECT sessions made the ratings. The CGI-I rating was performed within one week after ECT. When the nurses made the ratings, they had access to the charts including the doctors’ professional assessments and the patients’ self-assessments used in the routine care. In the analysis, CGI-I 1 or 2 was considered improved and 3 or 4 was considered not improved.

The severity of depression was assessed by the treating physician according to the International Classification of Diagnosis 10^th^ version (ICD) as mild/moderate, severe without psychosis or severe with psychosis. A corresponding severity classification was used in schizoaffective disorders, depressed type.

The outpatients were those with at least one ECT administered in an outpatient setting. Thus if the treatment was initiated in an inpatient setting and continued in an outpatient setting the patient was considered to be an outpatient in the statistical analysis.

The voluntary/involuntary hospital admission status variable was based on the legal status of the patient. In Sweden, verbal consent to ECT is standard and the treating physician can administer ECT without consent during involuntary care. Some voluntarily hospitalised patients may recognise that they have to accept ECT or else risk involuntary care. Some involuntarily hospitalised patients may consent to ECT.

### Validation of measures

In 524 patients CGI-I improvement could be compared to the pre to post ECT change in Global assessment of functioning – symptoms score (GAF-S). Patients improved according to CGI-I increased their GAF-S by 26 (SD 13) while those not improved increased their GAF-S score by 8 (SD 8). In 80 patients pre to post ECT change in Montgomery Åsberg Depression Rating Scale (MADRS) was available. Improved patients decreased MADRS by 26.6 (SD 12.5) and not improved by 6 (11.1). Change in Montgomery Åsberg Depression Rating Scale –Self Assessment (MADRS-SA) was available in 349 cases. Improved patients decreased MADRS-SA by 23.2 (SD 9.7) and not improved by 10.1 (SD 10.0). All measures were recorded within one week before ECT and within week after ECT. The nurses were trained to assess Clinical Global Impression-Improvement, but the inter-rater reliability was not investigated.

The severity classification according to ICD was compared to the Clinical Global Impression –Severity (CGI-S) score before ECT in 867 patients. CGI-S was assessed by the physician who referred the patient for ECT. On CGI-S patients classified as mild/moderately depressed were scored 4.2 (SD 0.6), severe without psychosis 5.2 (SD 0.7) and severe with psychosis 5.7 (SD 0.6). GAF-S scores before ECT was available in 601 cases. GAF-S scores were, mild/moderately depressed patients 44 (SD 10), severe without psychosis 39 (SD 11), severe with psychosis 33 (SD 11). MADRS scores amongst 151 patients before ECT were for mild/moderately depressed 30.3 (SD 8.6), severe depression without psychosis 34.0 (SD 6.8), severe depression with psychosis 36.4 (SD 7.9). MADRS-S scores amongst 465 patients before ECT were for mild/moderately depressed 31.7 (SD 7.9), for severely depressed patients without psychosis 35.5 (SD 7.4) and for patients with severe depression with psychosis 33.4 (SD 9.5).

### Statistics

Frequency distributions were tested by means of chi-square tests. Differences between means were tested by the Student's *t*-test.

To assess the relative importance of certain factors, a logistic regression, forward conditional, with improved as dependent variable and factors with a trend toward statistical significance in the univariate analysis entered (p < 0.10).

The tests performed were two sided and alpha was set to 0.05. SPSS version 15.0 (SPSS Inc, Chicago, Ill) was used for the statistical analyses.

## Results

### Responder rate

Out of 936 patients, 750 were improved according to CGI-I corresponding to an overall responder rate of 80.1%.

### Age, sex and diagnosis

A higher proportion of older patients (>50 years) responded (84.3%) as compared to younger patients (74.2%, p < 0.001). The responder rates were nearly exactly the same in men and women. Patients with severe, psychotic depressions had the highest responder rate (88.9%), as compared to patients with severe, non-psychotic depressions (81.5%) and patients with mild/moderate depressions (72.8%), p < 0.001). However, the responder rates were similar among patients suffering from bipolar I, bipolar II, major depressive disorder, single episode and major depressive disorder, recurrent depressions. There was a trend towards a lower responder rate (68.8%, p = 0.060, df = 4) amongst patients treated for a depressive episode of schizoaffective disorder. The lower responder rate amongst patients with schizoaffective disorder, depressed type was statistically significant in a post hoc comparison to all other patients (χ^2^ = 4.1, p = 0.04, df = 1). Patients with co-morbid anxiety disorders and dependence disorders had a similar responder rate as compared to patients without such diagnoses. Patients with personality disorders had a lower responder rate (66.2%) as compared to patients without co-morbid personality disorders (81.4%, p = 0.001) (Table 1).

**Table 1:** Proportion of patients responding (CGI-I = 1 or 2) to ECT in relation to patient characteristics

			
Sex N = 936	Men (n = 399)	79.7%	χ^2^ = 0.08 p = 0.78
	Women (n = 537)	80.4%	
Age (years) N = 936	Age < 50 years (n = 388)	74.2%	χ^2^ = 14.5, **p < 0.001**
	Age ≥ 50 years (n = 548)	84.3%	
Diagnoses N = 936	Major depression, single episode (n = 149)	85.2%	χ^2^ = 9.1 df, =4 p = 0.060
	Major depression, recurrent (n = 559)	81.0%	
	Bipolar I, depressed (n = 113)	77.9%	
	Bipolar II, depressed (n = 67)	73.1%	
	Schizoaffective disorder, depressed (n = 48)	68.8%	
Severity N = 917	Mild/moderate (n = 290)	72.8%	χ^2^ = 21.7 df 2 **p < 0.001**
	Severe, non-psychotic (n = 401)	81.5%	
	Severe, psychotic (n = 226)	88.9%	
Co-morbid Anxiety diagnosis N = 936	With co-morbid anxiety diagnosis (n = 254)	78.0%	χ^2^ = 1.04, p = 0.31
	Without co-morbid anxiety diagnosis (n = 682)	80.9%	
Co-morbid Dependence diagnosis N = 936	With co-morbid dependence diagnosis (n = 102)	77.5%	χ^2^ = 0.52, p = 0.47
	Without co-morbid dependence diagnosis (n = 834)	80.5%	
Co-morbid Personality disorder N = 936	With personality disorder (n = 77)	66.2%	χ^2^ = 10.2, **p = 0.001**
	Without known personality disorder (n = 859)	81.4%	
Out/in patient N = 936	Outpatient (n = 181)	66.3%	χ^2^ = 27.0, **p < 0.001**
	Inpatient (n = 755)	83.4%	
Involuntary admission N = 936	Involuntary hospital admission (n = 144)	87.5%	χ^2^ = 5.81, **p = 0.016**
	Voluntary hospital admission (n = 792)	78.8%	

### Treatment factors

Inpatients had an increased responder rate (83.4%) as compared to outpatients (66.3%, p < 0.001). Also involuntarily admitted patients had higher responder rate (87.5%) as compared to voluntarily (78.8%, p = 0.016) admitted patients. Furthermore, there were no significant differences in responder rates whether unilateral ECT, bifrontal ECT or bitemporal ECT was used, nor did the dosage at the last ECT differ between responders and non-responders (data not shown).

The number of ECT sessions were analysed in different subgroups. Older patients (>50 years) received 8.0 ECT (SD 3.0) and younger patients received 8.1 (SD 3.4). Mild/moderately depressed patients received 8.2 ECT (SD 3.3), severely depressed patient without psychosis 8.0 (3.1) and severely depressed patients with psychosis 7.8 ECT (SD 3.0). Patients treated for schizoaffective disorder, depressed type received 8.7 ECT (SD 3.1) compared to other patients 8.0 (SD 3.2) (p = 0.17). Patients with personality disorders received 8.6 ECT (SD 3.6) compared to 8.0 (3.1) for patients without personality disorder. Outpatients received 9.1 ECT (SD 4.1) and inpatients 7.8 (SD 2.8), the difference was statistically significant (p < 0.001).

### Significant predictors

Older age, absence of schizoaffective disorder, psychotic symptoms and inpatient status were independent significant predictors of response in a forward conditional logistic regression analysis. Improvement was the dependent variable and age, diagnosis, severity of depression and in/out patient status were independent variables.

The odds-ratios of the response rate increased with age in years (95% confidence interval 1.00-1.02). Patients treated for schizoaffective disorder, depressed type had a lower responder rate compared to patient treated for recurrent major depressive disorder (95% confidence interval of the odds-ratio 0.15-0.70). Mildly to moderately depressed patients and severely depressed patients had lower response rates compared to psychotically depressed patients (odds-ratios 0.22-0.63 and 0.30-0.89 respectively). As compared to inpatients, outpatients had a lower response rate (odds-ratio 0.32-0.70) (Table 2).

**Table 2:** Odds-ratio of response (CGI-I 1 or 2) in relation to independent variables: age, diagnosis, severity voluntary/involuntary hospital admission status and in/out patient status (forward conditional logistic regression) n = 917

		**Exp.(beta)**	**Confidence interval**	**Significance**
At last step	Outpatient	0.47	0.32-0.70	<0.001
	Inpatient	Reference	Reference	Reference
	Severity (mild/moderate)	0.37	0.22-0.63	<0.001
	Severity (severe non-psychotic)	0.52	0.30-0.89	0.017
	Severity (severe psychotic)	Reference	Reference	Reference
	Age	1.01	1.00-1.02	0.006
	Major depressive disorder, recurrent	Reference	Reference	Reference
	Major depressive disorder, single episode	1.16	0.69-1.94	0.58
	Bipolar I, depressive episode	0.80	0.48-1.36	0.41
	Bipolar II, depressive episode	0.68	0.37-1.24	0.21
	Schizoaffective disorder, depressive episode	0.32	0.15-0.70	0.004
		Model chi square = 58.6, df = 8, p < 0.001		

## Discussion

Four out of five patients in a consecutive clinical sample were improved by ECT similar to earlier reports from clinical trials [3,4] and from clinical routine [5].

More severe forms of depression were associated with higher proportion of responders to ECT, well in line with earlier studies [6]. It is difficult to explain why some earlier smaller studies have reported less positive outcome in patients with psychotic depression [8,9]. However, the result from this study is robust and the reported responder rate in psychotic depression is very high. The Swedish National Health and Welfare Board has given ECT the highest priority in patients with severe forms of depression [17]. Our results are in line with such a policy. The very high responder rate in psychotic depression calls for early consideration of ECT in such patients.

Older age was associated with a higher proportion of responders to ECT. Our study provides further support to earlier studies [7,10,12] indicating that older patients benefit more than younger patients from ECT. In addition, older patients may not tolerate the side effects of antidepressant drugs, especially not the anticholinergic effects of tricyclics. Therefore, ECT should be considered in geriatric depression.

Inpatient treatment was associated with a higher responder rate than outpatient treatment. This effect might be confounded by the severity of the disease. An attempt was done to correct for severity of disease with regression analysis. However, it is difficult to fully correct for severity as inpatients are so often more symptomatic than outpatients. Especially some symptoms such as psychosis, suicidal ideation and retardation may be more prevalent among inpatients. Such symptoms may be especially prone to respond to ECT. Outpatients had longer treatment series than inpatients, thus the difference could not be attributed to shorter treatment series for outpatients. More intensive supportive care may explain part of the difference. Also, inpatients are more closely observed and it may be easier to adjust the treatment according to the response.

The presence of a personality disorder has been reported to be associated with a lower responder rate after ECT [18] and other treatment modalities for depression [19]. It is a weakness of the study that no uniform structured interview was used to diagnose personality disorders and the condition may be unrecognised in clinical practice. The data support a reasonably high responder rate also amongst patients with apparent personality disorders. Patients with personality disorders should therefore not automatically be disqualified from receiving ECT, when suffering from psychotic or severe depression.

Patients treated for a depressive episode of a schizoaffective disorder had a lower responder rate as compared to patients with major depressive disorder and bipolar disorder. This result is based on a limited number of observations and is therefore somewhat uncertain.

Strengths of the study include the large consecutive population-based cohort and that the results reflect current clinical routine use of ECT in Sweden.

Weaknesses include that some factors that have been associated with response to ECT in earlier studies were not recorded such as duration of depressive symptoms in the episode and duration of pharmacologic treatment. Therefore we cannot control for these factors. Also, the presence of melancholic features was not recorded. The CGI-I inter-rater reliability was not assessed. Moreover, as CGI-I was performed by nurses involved with the treatments, there is a risk of over-estimating the response. The diagnoses were based on routine clinical procedures. Thus, the diagnoses (e.g. major depressive episode vs bipolar spectrum depression) should be considered clinical estimates as no uniform structured interview was used.

This study focuses on the outcome immediately following ECT and is not informative of the longer term outcome. Treatment response in this study is also not equal to complete symptomatic remission. Many patients have remaining symptoms after ECT that need to be addressed even though they are significantly improved. Thus, a high responder rate does not automatically mean a high remission rate. We have reported earlier that symptom reoccurrence is common soon after ECT [20]. Further studies are needed to describe the longer term outcome with ECT. We hope that the growing quality register will be able to contribute to that.

## Conclusions

Our results show that psychotically depressed patients have a very high probability of benefit from ECT. We further conclude that the responder rate to ECT tends to be high for all groups investigated. Even in the least responsive groups most patients responded to ECT. Furthermore, inpatient ECT may be more effective than outpatient ECT.

## Competing interests

The authors declare that they have no competing interests.

## Authors’ contributions

AN participated in the design and coordination of the study, participated in acquiring the data, analysed the data and drafted the manuscript. LvK participated in the design and coordination of the study, analysed some of the data and revised the manuscript. IE participated in the design and coordination of the study and revised the manuscript. All authors’ read and approved the final manuscript.

## Pre-publication history

The pre-publication history for this paper can be accessed here:

http://www.biomedcentral.com/1471-244X/12/115/prepub
